# Acadesine Triggers Non-apoptotic Death in Tumor Cells

**Published:** 2013

**Authors:** V. A. Glazunova, K. V. Lobanov, R. S. Shakulov, A. S. Mironov, A. A. Shtil

**Affiliations:** Blokhin Cancer Center, Russian Academy of Medical Sciences, 24, Kashirskoe shosse, Moscow, Russia, 115478; 2State Research Institute of Genetics and Selection of Industrial Microorganisms, 1, Dorozhny proezd, Moscow, Russia, 117545

**Keywords:** acadesine, cell death, tumor cells

## Abstract

We studied the cytotoxicity of acadesine (5-aminoimidazole-4-carboxamide-1-β-D-ribofuranoside) for tumor and normal cells of various species and tissue origin. In tumor cells, acadesine triggered non-apoptotic death; the potency of the compound to normal cells was substantially lower. Acadesine was toxic for tumor cells with multidrug resistant phenotypes caused by the transmembrane transporter Р-glycoprotein or lack of proapoptotic p53. Activity of adenosine receptors was required for acadesine-induced cell death, whereas functioning of АМР-dependent protein kinase was not required. A more pronounced cytotoxicity for tumor cells, as well as the non-canonical death mechanism(s), makes acadesine a promising candidate for antitumor therapy.

## INTRODUCTION

Acadesine (5-**aminoimidazole**-4-carboxamide-1-β-Dribofuranoside, AICAR) is currently undergoing clinical trials as an agent for treating chronic lymphocytic leukemia [1, 2]. A very important property of acadesine is its preferential toxicity for tumor cells, while nontumor cells are damaged to a lesser significant extent [2, 3]. It has been demonstrated previously that acadesine can stimulate AMP-activated protein kinase (AMPK), the essential regulator of the cellular energy balance that controls the oxidation of fatty acids, the glucose metabolism, and synthesis of proteins, fatty acids, and cholesterol [4-10]. The mechanism of action of acadesine is determined by its phosphorylation with adenosine kinase, yielding ZMP (5-amino-4-imidazole carboxamide ribotide), an intermediate product of de novo synthesis of purine nucleotides [1, 4, 5, 8]. ZMP can activate AMPK by imitating the metabolic effects of AMP. The antitumor effect of acadesine is attributed to apoptosis induction [7, 9, 11, 12]. Meanwhile, data on non-apoptotic cell death and the AMPK-independent mechanism of action of acadesine on tumor cells have also been obtained [12, 13].

The effect of acadesine on mammalian cells was studied in this work. Acadesine was shown to trigger the death of tumor cells of different tissue origins, including those resistant to a number of antitumor agents. The mechanisms of cell death differ from apoptosis; the necessity for adenosine transport turns out to be their crucial feature. The selectivity of the cytotoxic effect and features of the mechanisms of tumor cell death may be significant factors that determine the potential use of acadesine in antitumor therapy.

## EXPERIMENTAL

The following human cell lines were used in the experiments: НСТ116 (large intestine adenocarcinoma), НСТ116р53КО (isogenic p53 knockout subline), К562 (promyelocytic leukemia), К562/4 (subline obtained after selection for survival in the presence of doxorubicine; the multidrug resistance protein (MDR) P-glycoprotein (Pgp) was expressed), MCF-7 (breast adenocarcinoma), MCF-7Dox (subline obtained after selection for survival in the presence of doxorubicine; Pgp-mediated MDR phenotype), passaged human fibroblasts 2 (PHF-2), lymphocytes from healthy blood donors, and murine cells P388 (lymphocytic leukemia) and Sp2/0 (myeloma). The reagents were purchased from Pan- Eco (Russia), except for the specially mentioned cases. The cells were grown in Dulbecco’s modified Eagle’s medium (DMEM) supplemented with 5% fetal bovine serum (BioWhittaker, Austria), 2 mM *L*-glutamine, 100 AU/ml penicillin, and 100 μg/ml streptomycin at 37°С, 5% СО_2_ in a moist atmosphere. Cultures in the logarithmic growth phase were used for the experiments. Lymphocytes were isolated from the peripheral blood from donors via centrifugation in a ficoll–urographin density gradient (*d *= 1.077 g/cm^3^). ;

**Table 1:** Acadesine cytotoxicity for mammalian cells

Cells	Acadesine, mM					
	0	0.125	0.25	0.5	1.0	2.0
К562	100*	100	70	46	9	0
P388	100	36	30	20	9	0
Sp2/0	100	34	29	14	0	0
К562/4	100	100	72	42	8	0
MCF-7	100	100	82	50	15	2
MCF-7Dox	100	100	86	48	17	1
HCT 116	100	100	50	36	23	0
HCT 116p53KO	100	100	54	34	25	0
PHF-2, proliferating	100	100	100	96	96	86
PHF-2, nonproliferating	100	100	100	100	95	92
Donor lymphocytes	100	100	100	98	94	90

Note. MTT assay data of the cells after 72 h of incubation are shown. *The survival rate of the cells incubated without acadesine were taken as 100%. Each value is the average value of five independent experiments; standard deviation ≤ 0%. ** Fibroblast proliferation was terminated by growing cells until the monolayer reached 100% confluency (contact inhibition of cell division).

Acadesine was obtained at the State Research Institute of Genetics and Selection of Industrial Microorganisms via the microbiological procedure using an original recombinant strain [14]. Moreover, the cytotoxicity of acadesine purchased from Sigma was assessed. Dipyridamole (inhibitor of adenosine receptors) [8], 5-iodotubercidine (adenosine kinase inhibitor preventing the conversion of acadesine to ZMP), and zVAD-fmk (carbobenzoxyvalylalanyl-aspartyl-[O-methyl]-fluoromethylketone), a pan-caspase inhibitor, were also purchased from Sigma. All the compounds were dissolved in dimethyl sulfoxide or water (10–20 mM) and stored at –20°С. On the day when the experiment was supposed to take place, dilutions of the sample in the culture medium were prepared. The MTT assay, staining with propidium iodide and Annexin V conjugated to fluorescein isothiocyanate (FITC ), determination of the cell cycle by flow cytofluorometry, and electrophoretic analysis of the integrity of genomic DNA were used to assess acadesine cytotoxicity [15, 16]. An apoptosis inductor, alkyl cationic glycerolipid *rac-*N- {4-[(2-ethoxy-3-octadecyloxy)prop-1-yloxycarbonyl] butyl}-N*’*-methylimidazolium iodide, was used as the control compound in individual experiments [17].

## RESULTS AND DISCUSSION

**Predominant sensitivity of tumor cells to acadesine**

We had ascertained in the preliminary experiments that an acadesine sample obtained microbiologically and commercial acadesine are characterized by identical physicochemical properties, purity, storage stability, and cytotoxicity (data not shown). Acadesine obtained according to the authors’ procedure was used for further experiments. *Table 1* lists the cytotoxicity of acadesine for the transformed and non-transformed cells (cultured or freshly isolated) originating from different species and tissues.

It follows from the data listed in *Table 1*that P388 (murine leukemia) and Sp2/0 (murine myeloma) cells exhibit the highest sensitivity to acadesine: ~ 1/3 of the cell population survives at an acadesine concentration of 0.125 mM. Sub-millimolar concentrations of acadesine also cause the death of other transformed cell lines. It is important to note that the acadesine cytotoxicities are almost identical for the K562 leukemia cell line and its subline with Pgp-mediated MDR (K562/4). This is also valid for a MCF-7 breast adenocarcinoma cell line and the MDR subline (*Table 1*). The comparison of the cytotoxicities of acadesine for a HCT 116 line and HCT 116p53KO subline (resistant to a number of DNAdamaging anticancer drugs) [18] has demonstrated that inactivation of the proapoptotic protein p53 does not increase the survival rate of cells in the presence of acadesine.

**Fig. 1:**
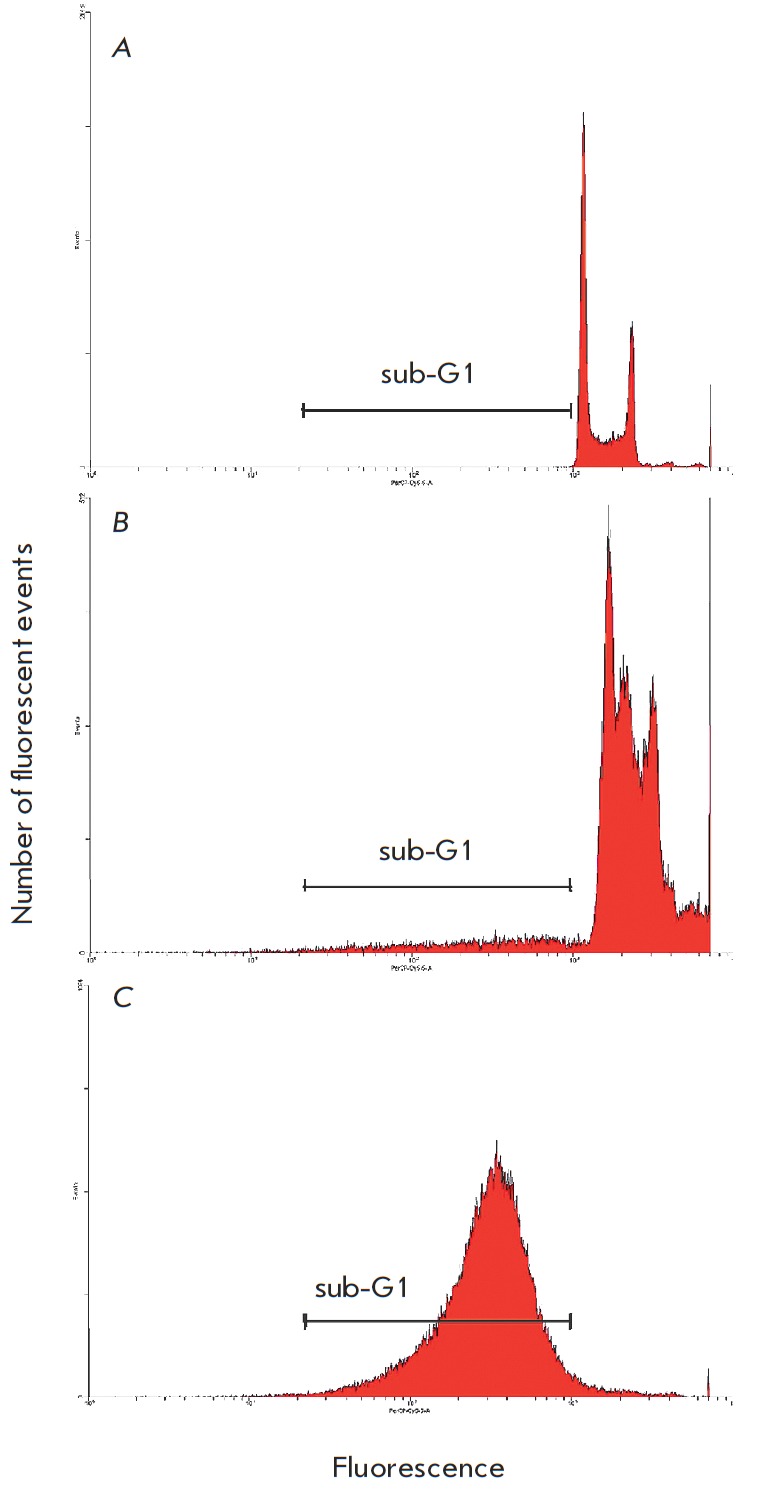
Cell cycle distribution of HCT116 cells treated with 0.4 mM acadesine. A – intact cells; B – arrest in S phase after 24 h; C – sub-G1 peak after 48 h

The considerably higher survival rate of nontumor cells in the presence of acadesine is also important: death of the donor’s lymphocytes and non-transformed fibroblasts was virtually absent even when continuously exposed to acadesine at millimolar concentrations for 72 h (*Table 1*). Thus, acadesine primarily causes the death of transformed cells (suspension and epithelial ones), including the sublines resistant to other anticancer drugs. Nontumor cells are damaged by acadesine to a significantly lesser extent. These features speak to the potential of using acadesine as an antitumor agent. However, the mechanisms that underline the toxicity of acadesine for tumor cells need to be understood.

**Acadesine causes non-apoptotic cell death**

**Fig. 2:**
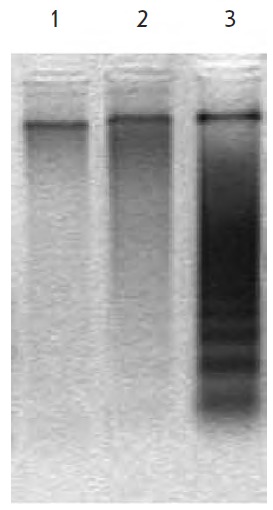
DNA integrity in HCT116 cells. 1 – intact cells; 2 – acadesine, 0.4 mM, 24 h; 3 – alkyl cationic glycerolipid [17], 6 μM, 24 h (technical control)

The effect of acadesine on ploidy distribution in a HCT 116 large intestine adenocarcinoma cell line was studied by flow cytofluorometry. Arrest in the S phase and massive cell death (the region to the left of the G1 peak; hypodiploid nuclei) (*Fig. 1*) were observed 24 and 48 h, respectively, after the introduction of acadesine (0.25 mM). Accumulation of fragmented DNA can be indicative of apoptotic cell death if the DNA is split in internucleosomal regions, which can be seen from the formation of a number of 140- to 170-bp-long fragments during electrophoresis. In order to verify this idea, DNA integrity in acadesine-treated HCT 116 cells was determined. It turned out that acadesine, as opposed to the control compound (alkyl cationic glycerolipid [17]), does not result in the emergence of a DNA ladder typical of apoptosis (*Fig. 2*).

**Fig. 3:**
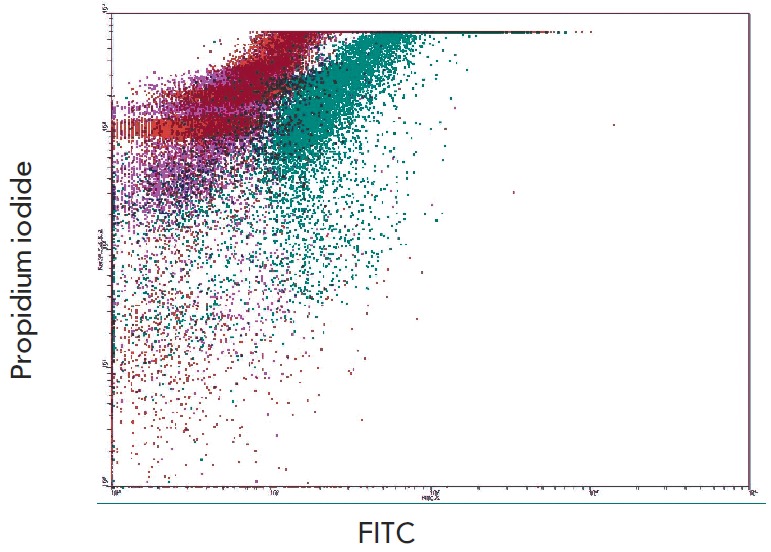
Staining of HCT116 cells with Annexin V-FITC and propidium iodide. Pseudo colors: red – intact cells; violet – acadesine (0.4 mM, 24 h); blue – alkyl cationic glycerolipid (see legend to Fig. 2)

The results of staining cells with Annexin V-FITC and propidium iodide (*Fig. 3*) argue in favor of a nonapoptotic mechanism of death of НСТ116 cells under the action of acadesine. Annexin V binds to phosphatidylserine on the plasma membrane (translocation of phosphatidylserine from the inner lipid layer of the membrane to the outer one is considered to be a sign of apoptosis). Propidium iodide is capable of penetrating into cells undergoing necrosis (disrupting the integrity of the plasma membrane). Acadesine-treated НСТ116 cells (0.4 mM, 24 h) were not stained with Annexin V-FITC ; contrariwise, the cells accumulated propidium iodid (*Fig. 3*), which allows one to hypothesize about a necrotic component of the cell death mechanism. Similar results were obtained when necrotic cells were visualized using trypan blue (data not shown). The disruption of the integrity of the plasma membrane is presumably a late event during acadesine-induced cell death. The control agent, alkyl cationic glycerolipid, caused an increase in the number of Annexin V-positive cells, which is typical of apoptosis (*Fig. 3*). Since apoptotic cell death assumes that caspases play a significant role in it, the effect of the pan-caspase inhibitor zVAD-fmk on acadesine cytotoxicity was studied. HCT 116 cells were incubated with 200 μM zVAD-fmk for 30 min; acadesine was then introduced in the cultures, and the incubation was carried out for an additional 24 h. The presence of zVAD-fmk did not reduce cell death, which supports the conclusion about the non-apoptotic mechanism of the cytotoxicity of acadesine.

**Interaction with the adenosine receptors is required to ensure the death of tumor cells under the action of acadesine**

Acadesine can be transferred from the extracellular space into the cells by adenosine transporters [19]. We have studied the effect of dipyridamole, the inhibitor of these transporters, on acadesine cytotoxicity in a P388 cell line. Cells turned out to be insensitive even to the relatively high (up to 0.8 mM) acadesine concentrations in the presence of dipyridamole (*Table 2*).

**Table 2:** Acadesine cytotoxicity in combinations with dipyridamole or 5-iodotubercidine

Effect	Acadesine, mM					
	0	0.08	0.1	0.2	0.4	0.8
Acadesine	100*	79	38	33	20	18
Acadesine + dipyridamole, 5 μM	100	100	99	99	100	101
Acadesine + 5-iodotubercidine, 0.05 μM/0	100	76	39	31	22	16

*Survival rate (%) of P388 leukemia cells according to the MTT assay data after 72 h of incubation.

In order to shed light on the role of the metabolic pathway acadesine–ZMP–AMPK in the cytotoxicity of acadesine (its phosphorylation by adenosine kinase yielding ZMP and activation by AMPK), cells were incubated with acadesine and the adenosine kinase inhibitor 5-iodotubercidine. The inhibitor had no effect on acadesine cytotoxicity (*Table 2*). Hence, cell death in response to acadesine is not caused by the formation of ZMP or activation of AMPK.

Thus, the investigation into the mechanisms of acadesime cytotoxicity has revealed a number of features indicating a nontrivial nature of the pharmacological effects of this compound. Acadesine triggers death in cultured tumor cells, while its effect on nontumor cells is pronounced to a considerably lesser extent. Acadesine is toxic for cells with molecular determinants of drug resistance: Pgp expression and non-functional p53. It is important to emphasize the non-apoptotic character of the tumor cell death induced by acadesine. These results provide grounds for regarding acadesine as a crucial agent for studying the mechanisms of tumor cell death and a promising drug candidate.

The question regarding the intracellular target of acadesine, the interaction with which causes tumor cell death, remains open. We have demonstrated that the function of adenosine transporters is the criterion for cell death, while no activation of AMPK is required. It is reasonable to assume that tumors expressing the aforementioned adenosine transporters and receptors will exhibit the highest sensitivity to acadesine. The role of the purine nucleotide transport in cell death has yet to be studied sufficiently; an analysis of the differential expression of adenosine carriers and receptors in different types of tumors is required. The enhanced expression of these molecules may turn out to be a novel molecular marker of tumor sensitivity to acadesine and a criterion for the selection of patients for proper therapy.
